# Acculturation and other risk factors of depressive disorders in individuals with Turkish migration backgrounds

**DOI:** 10.1186/s12888-017-1430-z

**Published:** 2017-07-19

**Authors:** Hanna Janssen-Kallenberg, Holger Schulz, Ulrike Kluge, Jens Strehle, Hans-Ulrich Wittchen, Uwe Wolfradt, Uwe Koch-Gromus, Andreas Heinz, Mike Mösko, Demet Dingoyan

**Affiliations:** 1 Department of Medical Psychology, Study group on Psychosocial Migration Research, University Medical Centre Hamburg-Eppendorf, Martinistraße 52, Building W(est)26, 20246 Hamburg, Germany; 2 Department of Psychiatry and Psychotherapy, Charité – Universitätsmedizin Berlin, Charitépl. 1, 10117 Berlin, Germany; 3 Berlin Institute for Integration and Migration Research, Department Migration, Mental and Physical Health and Health Promotion, Faculty of Humanities and Social Sciences, Humboldt University Berlin, Unter den Linden 6, 10099 Berlin, Germany; 4 Institute of Clinical Psychology and Psychotherapy, Technische Universität Dresden, Chemnitzer Straße 46, 01187 Dresden, Germany; 5 Institute of Psychology, Martin Luther University Halle-Wittenberg, Emil-Abderhalden-Str. 26-27, 06108 Halle (Saale), Germany

**Keywords:** Depression, Turkish, Prevalence, Acculturation, Risk factors, Migration

## Abstract

**Background:**

Acculturation is a long-term, multi-dimensional process occurring when subjects of different cultures stay in continuous contact. Previous studies have suggested that elevated rates of depression among different migrant groups might be due to patterns of acculturation and migration related risk factors. This paper focused on prevalence rates of depressive disorders and related risk factors among individuals with Turkish migration backgrounds.

**Methods:**

A population-based sample of 662 individuals with Turkish migration backgrounds were interviewed by bilingual interviewers using a standardised diagnostic interview for DSM-IV-TR and ICD-10 diagnoses (CIDI DIA-X Version 2.8). Associations between 12-month prevalence rates of depressive disorders with potential risk factors were assessed, including gender, age, socioeconomic status, acculturation status and migration status.

**Results:**

12-month prevalence rates of any depressive disorder were 29.0%, 14.4% of major depressive disorder (MDD) and 14.7% of dysthymia. Older age and low socioeconomic status were most consistently related to higher risks of depressive disorders. Acculturation status showed associations with subtypes of depressive disorder. Associations differed between men and women. Symptom severity of MDD was linked to gender, with females being more affected by severe symptoms.

**Conclusion:**

The prevalence of depressive disorders is high in individuals with Turkish migration backgrounds, which can be partly explained by older age, low socioeconomic status and acculturation pressures. Only a limited number of risk factors were assessed. Acculturation in particular is a complex process which might not be sufficiently represented by the applied measures. Further risk factors have to be identified in representative samples of this migrant group.

## Background

The number of migrants in Europe has increased rapidly over the last few decades, nearly two thirds of all migrants worldwide live in Europe or Asia [1]. With a total number of 12 million people Germany and the Russian Federation host the second largest migrant populations in the world [1, 2]. Equalling almost 3 million people, individuals with Turkish migration backgrounds constitute the largest migrant group in Germany and are defined as all Turks who immigrated to Germany after 1949 as well as all Turks born in Germany and all individuals born in Germany as Germans with at least one Turkish parent [2]. In response to labour shortages in the 1960s, Western Germany invited a large number of migrants from Turkey and other Mediterranean countries, who were often followed by their families later on [3].

## Migration and mental health

Research studies in US and Canada have provided evidence that the ‘healthy migrant effect’ not only holds for physical health but also for mental health [4, 5]. In line with these findings, American and Canadian studies concerning mood disorders in particular reported that migrants soon after immigration typically showed lower rates of mood disorders compared to the host population [6–8]. In contrast, European studies, found higher prevalence rates of depression in a substantial part of migrant groups compared to the host populations [9, 10]. A meta-analysis found combined prevalence rates of depression to be 20% (95% CI = 14–26) among labour migrants in Europe [11]. Elderly European migrants in particular showed a higher risk of depression relative to the host population [12, 13].

A possible consequence of migration is the long lasting and multi-dimensional process of acculturation defined as any change that occurs when individuals or groups from different cultures continuously stay in contact with each other [14–16]. One of the most widely studied acculturation concepts is Berry’s model of four different acculturation strategies (integration, assimilation, separation and marginalisation), based on two main components: maintenance of culture of origin and participation in the host culture [16, 17]. Other frequently used measures of acculturation were nativity, length of residency in the host country and language proficiency [18, 19]. An orientation towards both cultures, showed the most favourable effect on mental health and was negatively associated with depression [20, 21]. A lower risk of depression in foreign-born migrants relative to their native-born descendants was observed across different American migrant groups illustrating the difference between migrant generations [5, 7, 8, 22–24]. Early age at immigration as well as longer residency in the host country correlated with high risks of mood disorders among migrants [6, 8, 22, 25], whereas a study among Asian Americans found varying associations between depression and immigration-related variables, emphasising the need to differentiate between gender [23].

### Depressive disorders among individuals with Turkish migration backgrounds

Several large population-based studies in the Netherlands, Belgium and Germany demonstrated higher prevalence rates of depression in Turkish migrants of the first and second migration generation compared to the host population and other migrant groups [26–31]. Results of clinical research showed that depression is one of the most frequently diagnosed conditions in patients with Turkish origins in Germany, occurring more frequently and with a significantly higher severity relative to patients of the host-society [32, 33]. Other health care and clinical studies reported increased psychological distress among patients with Turkish migration backgrounds in Germany, particularly in female ones [34–36]. The probability of receiving treatment for unipolar depression was higher in Turkish migrants and their native-born descendants than in any other migrant group or the host population in the Netherlands [37].

Common risk factors related to elevated rates of depressive disorders among first and second generation migrants with Turkish origins were: being female [28, 29, 38], older age [29], and low socioeconomic status [26, 27, 29]. Regarding acculturation, integration was found to be the most beneficial acculturation strategy associated with low rates of depression in Turkish migrants of the first and second migration generation, whilst marginalisation and separation were associated with increased depression rates [38, 39]. When it comes to migration status, a higher risk and more severe symptoms of depression in foreign-born Turkish migrants of the first generation relative to their descendants born in the host country were reported [27, 31, 38].

### Aim of the study

To our knowledge epidemiological data of mental disorders among individuals with migration backgrounds is scarce in Germany, in particular regarding specific migrant groups and the use of standardised diagnostic instruments [40, 41]. The aim of this paper was to provide epidemiological data of depressive disorders among individuals with Turkish migration backgrounds in Germany. We focused on the 12-months prevalence of any depressive disorder, respectively MDD and dysthymia, as well as symptom severity of MDD. We also examine the following previously identified risk factors for depression: gender, age and socioeconomic status and explore the relationship with acculturation and migration status using the following constructs: cultural identity, mother tongue, language proficiency, nativity, migration generation, length of residency in Germany, age at immigration and citizenship.

## Methods

The study was part of an international research project‚ ‘Orientation of the health care system towards the needs of migrants with mental disorders‘, a co-operation between the University Medical Centre Hamburg-Eppendorf and the Charité-Universitätsmedizin Berlin. The study was approved by the Ethics committees of the Hamburg Chamber of Psychotherapists and the Ethics Commission and Data Commissioner of the Charité-Universitätsmedizin Berlin. An extensive description of the study design and sampling methods can be found in the study protocol [42] and is briefly summarised as follows.

### Sample

A total number of 662 standardized clinical interviews were completed in Hamburg (*n* = 376) and Berlin (*n* = 286). Participants met the following criteria: individuals with Turkish migration backgrounds, living in Berlin or Hamburg, aged from 18 to 65 years, consented to a face-to-face interview and had sufficient mobility to visit one of the interview offices in central locations.

#### Recruitment procedure and interview setting

The data collection of the present study took place in Hamburg and Berlin from August 2011 to July 2012. In order to increase the willingness of participation, focus groups, which aimed to identify potential recruitment barriers and resources, were conducted in a pre-study [43]. Study participants were recruited via a random sampling of the regional population register in districts of Hamburg which had a high percentage and density of individuals with Turkish migration backgrounds. The recruiting phase was accompanied by a public media campaign. In Hamburg, 10,873 individuals with Turkish citizenship or German citizenship and Turkish origin (i.e. due to a naturalisation) were identified through their citizenship status or by the onomastic procedure, which was based on a proven name-algorithm [44]. Potential participants were initially contacted via mail. Due to low response rates (on average 2.5%), snowball sampling was additionally applied in the last phase of data collection. In Berlin, a random sampling of individuals with Turkish migration background was not possible due to privacy protection laws. Participants were recruited directly by on-site collection at public locations, which had a high percentage and density of individuals with Turkish migration backgrounds and snowball sampling. During the snowball sampling in Hamburg and the data collection in Berlin, a quota scheme was applied originating from population-based micro census data of 2009, which contained the variables: sex (male/female), age (18–29/30–49/50–65) and education level (high/middle/low) to approximate a representative sample. Information and survey materials were available in both languages, Turkish and German. All interviews were conducted face-to-face by trained bilingual interviewers in one of the interview offices. Based on the participant’s language preference 458 of the completed interviews were conducted in Turkish language and 204 in German language.

The participants received an incentive of 10 Euro per 60 min of interviewing, as a gift card in Hamburg and cash in Berlin. The average length of an interview was 117 min.

### Measures

#### Depressive disorders

Depressive disorders were assessed by Section E of the CIDI DIA-X Version 2.8, a computer-assisted version of the ‘Diagnostic Expert system for disorders/Munich Composite International Diagnostic Interview’ (DIA-X/M-CIDI; Wittchen and Pfister, 1997). The CIDI DIA-X is a fully standardised, clinical face-to-face interview which assesses diagnoses of mental disorders along with symptom severity according to the international classification systems DSM-IV-TR with ICD-10 compatible codes [45]. It includes 109 questions to assess mood disorders. The CIDI DIA-X Version 2.8 was translated into Turkish according to Harkness [46] and was tested in a pre-trial. Quantitative and qualitative analyses support a comparable quality and feasibility level of the Turkish version of the instrument in contrast to the German version [47]. For a detailed description of the translation and editing process as well as the feasibility analysis of the translated CIDI DIA-X Version 2.8 review Dingoyan et al. [47]. The reliability of the M-CIDI for mood disorders was good with kappa values of 0.65 or above [48].

#### Sociodemographic data

The instrument contained 50 core questions on the basis of the sociodemographic module questions of the German Health Interview and Examination Survey for Adults [49] as well as the micro census 2010. Relevant sociodemographic measures for the present paper were gender, age and socioeconomic status measured by educational level and equivalent disposable household income. The equivalent disposable household income was calculated according to the modified Organisation for Economic Cooperation and Development (OECD) equivalence scale, taking into account the size of the household and age of its members [50]. A 1.0 weight was assigned to the head of the household, every additional household member received a weight of 0.5 and children up to 15 years of age received a weight of 0.3. The total monthly disposable income was divided by the sum of the household members’ weights to obtain the equivalent disposable household income [50].

#### Acculturation and migration status

Measures of acculturation status were added to the instrument for sociodemographic data. Language proficiency was used as a proxy measure of acculturation (maintenance of culture of origin and/or participation in the host culture), which was measured by the migrants’ self-determined ability to speak German and/or Turkish and the mother tongue. Another indicator was perceived cultural identity, with participants being asked which term they would use to label themselves regarding their cultural identity. Migration status was assessed by nativity, migration generation, length of residency in Germany, age at immigration and citizenship.

### Statistical analysis

Data analysis was conducted by SPSS Statistics version 22. Cross-tabulations were performed to calculate 12-month prevalence rates for each risk factor. Values were rounded to one decimal place. The total number of cases differs by variable considered caused by missing values.

A series of stepwise bivariate logistic regressions was conducted in order to assess the association between risk factors and prevalence rates of depressive disorders. Odds ratio (OR) and 95% confidence intervals (CI) were also computed. The significance of individual risk factors was assessed by the Wald-test. Likelihood ratio tests were conducted for the overall fit of the different models and the values of Nagelkerke R^2^ were reported [51]. Cross tables were calculated and Fisher’s exact test was performed to explore the relationship between symptom severity and risk factors. Due to a lack of variability in the data, categories of citizenship, cultural identity, mother tongue and language proficiency were partly merged together in order to perform stepwise bivariate logistic regression analyses. Additionally, the variables nativity, migration generation and age at immigration had to be excluded from the analyses because of redundancies in the data pattern. They were indirectly represented by length of residency. In view of the fact that gender is one of the most common risk factors for depressive disorders, results were presented separately for male and female participants. A differentiation of the two migration generations had to be forwent given the large differences in numbers of participants belonging to the first and second generation.

## Results

The characteristics of the study sample and the 12-month prevalence rates of any depressive disorder (MDD or dysthymia), MDD and dysthymia by risk factors are presented in Table 1. Group sizes were relatively balanced for sociodemographic data due to the sampling procedure. The majority of the sample described themselves as Turkish or German-Turkish. Only1.4% of participants described themselves as having a German cultural identity, even though 28.4% of participants held German citizenship. Over 80% declared Turkish to be their mother tongue, however almost all participants claimed to speak German and Turkish. Over three quarters of the sample were migrants of the first generation, originating from Turkey.

**Table 1:** Study sample characteristics and 12-month prevalence rates of depressive disorders by risk factors

	Frequency		Any depressive disorder	MDD	Dysthymia
	*n*	%	%	%	%
Total	662	100	29.0	14.4	14.7
Gender					
Male	276	41.7	23.2	10.5	12.7
Female	386	58.3	33.2	17.1	16.1
Age, y					
18–29	139	21.0	22.3	11.5	10.8
30–49	385	58.2	27.3	14.3	13.0
50–65	138	20.8	40.6	17.4	23.2
Education					
Low	257	38.8	35.4	14.8	20.6
Moderate	161	24.3	30.4	17.4	13.0
High	244	36.9	21.3	11.9	9.4
Income, €					
≤ 921	360	65.2	33.1	15.0	18.1
922–1417	117	21.2	25.6	11.1	14.5
> 1418	75	13.6	21.3	14.7	6.7
Cultural identity					
Turkish	295	45.7	28.1	12.5	15.6
German	9	1.4	33.3	22.2	11.1
German-Turk^a^	188	29.1	30.9	17.0	13.8
Other identity	153	23.7	28.8	14.4	14.4
Mother tongue					
Turkish	534	81.8	30.1	15.5	14.6
German	17	2.6	23.5	5.9	17.6
Both	63	9.6	23.8	11.1	12.7
Other language	39	6.0	25.6	10.3	15.4
Language proficiency					
Turkish	61	9.3	34.4	14.8	21.3
German	4	0.6	25.0	25.0	0.0
Both	590	90.1	28.5	14.4	14.1
Nativity					
Turkey	502	76.9	30.7	15.3	15.3
Germany	151	23.1	24.5	11.9	12.6
Migration generation					
1st generation	502	76.9	30.7	15.3	15.3
2nd generation	151	23.1	24.5	11.9	12.6
Length of residency, y					
German-born	151	23.7	24.5	11.9	12.6
**≤** 10	59	9.2	27.1	10.2	16.9
10–20	124	19.4	27.4	17.6	15.3
21–30	102	16.0	31.4	18.3	13.7
> 30	202	31.7	34.2	11.9	15.8
Age at immigration, y					
German-born	151	23.7	24.5	11.9	12.6
**≤** 13	118	18.5	34.7	17.8	11.9
13–18	118	18.5	29.8	19.5	15.3
18–25	148	23.2	27.0	24.2	16.9
> 25	103	16.1	29.8	10.7	17.5
Citizenship					
Turkish	415	63.5	28.4	13.7	14.7
German	184	28.4	32.8	18.3	14.5
Both	53	8.1	22.6	7.5	15.1

^a^person with Turkish migration background

Women, older people and people with low socioeconomic status in particular were affected by higher prevalence rates of depressive disorders. Concerning acculturation factors subjects with a German cultural identity suffered more frequently form depressive disorders, while individuals who reported Turkish as their single mother tongue and who only spoke Turkish showed elevated prevalence rates of depressive disorders. Migrants of the first generation were more at risk of suffering from depressive disorders compared to the native-born descendants, in particular those who immigrated under the age of 13 and those who had stayed over 30 years in Germany.

In order to assess the association of sociodemographic-related (Model 1), acculturation-related (Model 2) and migration-related (Model 3) risk factors with any depressive disorder, MDD and dysthymia ORs are represented in Tables 2, 3 and 4. Table 2 shows that older age represented the strongest independent risk factor for any depressive disorder. Male (*p* < .05) and female participants (*p* < .05) of older age were at a significantly higher risk to suffer from any depressive disorder than young individuals, even when adjusted for acculturation status and migration status. A low income showed a significant relationship with increased prevalence of any depressive disorder in male individuals, when controlled for acculturation and migration status (*p* < .05).

**Table 2:** Risk factors of 12-month prevalence rates of any depressive disorder – OR and 95% CI

	Any depressive disorder					
	Model 1		Model 2		Model 3	
	Men	Women	Men	Women	Men	Women
Age, y						
18–29	referent	referent	referent	referent	referent	referent
30–49	1.37 (0.53–3.79)	1.78 (0.91–3.49)	1.78 (0.66–4.83)	1.79 (0.91–3.53)	2.51 (0.81–7.74)	2.08 (0.97–4.49)
50–65	2.64 (0.95–7.33)	2.61* (1.18–5.81)	3.02* (1.01–9.00)	2.55* (1.14–5.72)	6.35* (1.48–27.23)	2.77* (1.03–7.49)
Education						
High	referent	referent	referent	referent	referent	referent
Moderate	1.26 (0.55–2.90)	0.1.60 (0.82–3.12)	1.09 (0.46–2.56)	1.55 (0.79–3.05)	1.32 (0.53–3.24)	1.58 (0.78–3.19)
Low	1.09 (0.50–2.38)	0.1.75 (0.96–3.19)	1.16 (0.52–2.60)	1.73 (0.93–3.21)	1.52 (0.64–3.62)	1.86 (0.96–3.60)
Income, €						
> 1418	referent	referent	referent	referent	referent	referent
922–1417	1.53 (0.46–5.07)	0.70 (0.27–1.80)	1.59 (0.46–5.45)	0.67 (0.26–1.76)	1.51 (0.42–5.38)	0.68 (0.26–1.79)
**≤** 921	2.73 (0.95–7.81)	0.83 (0.37–1.89)	2.87 (0.98–8.42)	0.80 (0.35–1.84)	3.36* (1.10–10.27)	0.91 (0.38–2.17)
Cultural identity						
Turkish			referent	referent	referent	referent
German, German-Turk^a^			1.40 (0.62–3.19)	1.23 (0.71–2.13)	1.57 (0.66–3.73)	1.09 (0.61–1.94)
Other identity			1.57 (0.67–3.69)	1.03 (0.52–2.03)	1.72 (0.70–4.23)	0.99 (0.49–2.00)
Mother tongue						
Turkish			referent	referent	referent	referent
German, both			1.47 (0.58–3.73)	0.80 (0.33–1.96)	1.44 (0.53–3.89)	0.75 (0.30–1.87)
Other language			0.22 (0.03–1.79)	0.84 (0.29–2.44)	0.19 (0.02–1.62)	0.84 (0.28–2.52)
Language proficency						
Turkish			referent	referent	referent	referent
German, both			1.65 (0.17–2.48)	0.86 (0.41–1.81)	1.94 (0.23–3.92)	0.80 (0.37–1.75)
Length of residency, y						
German-born					referent	referent
**≤** 10					2.46 (0.666–9.13)	1.03 (0.18–5.78)
10–20					0.62 (0.18–2.16)	1.85 (0.60–5.75)
21–30					0.85 (0.24–2.94)	1.65 (0.54–5.06)
> 30					0.36 (0.11–1.16)	2.04 (0.69–6.02)
Citizenship						
Turkish					referent	referent
German, both					1.41 (0.62–3.19)	1.61 (0.90–2.90)

^a^or person with Turkish migration background. *p < 0.05. Model fit:

Model 1: LR _male_ = 11.17(6), *p* = .083; *R* ^*2*^_male_ = .08, LR _female_ = 13.09(6), *p* < 0.05; *R* ^*2*^_*female*_^=^ .06,

Model 2: LR _male_ = 16.23(11), *p* = .133; *R* ^*2*^_male_^=^ .11, LR _female_ = 14.05(11), *p* < 0.05; *R* ^*2*^_*female*_^=^ .06,

Model 3: LR _male_ = 24.14(16), *p* = .088; *R* ^*2*^_male_ = .16, LR _female_ = 18.50(16), *p* = .296; *R* ^*2*^_*female*_ = .08

**Table 3:** Risk factors of 12-month prevalence rates of MDD – OR and 95% CI

	MDD					
	Model 1		Model 2		Model 3	
	Men	Women	Men	Women	Men	Women
Age, y						
18–29	referent	referent	referent	referent	referent	referent
30–49	1.13 (0.33–3.93)	2.25 (0.92–5.54)	0.83 (0.23–3.03)	2.49 (1.00–6.22)	0.68 (0.15–3.18)	2.34 (0.83–6.57)
50–65	1.40 (0.35–5.60)	2.61 (0.92–7.45)	0.98 (0.23–4.17)	2.78 (0.95–8.17)	0.64 (0.8–4.77)	2.26 (0.62–8.29)
Education						
High	referent	referent	referent	referent	referent	referent
Moderate	2.80 (0.82–9.65)	1.24 (0.57–2.68)	2.85 (0.77–10.52)	1.16 (0.52–2.60)	3.27 (0.87–12.34)	1.16 (0.51–2.60)
Low	1.71 (0.51–5.74)	0.88 (0.42–1.88)	1.61 (0.47–5.53)	0.85 (0.39–1.88)	1.87 (0.52–6.78)	0.89 (0.39–2.06)
Income, €						
> 1418	referent	referent	referent	referent	referent	referent
922- 1417	0.51 (0.15–1.71)	0.82 (0.37–1.84)	0.56 (0.16–1.92)	0.78 (0.34–1.76)	0.53 (0.15–1.86)	0.70 (0.30–1.63)
**≤** 921	0.32 (0.07–1.52)	2.18 (0.89–5.34)	0.32 (0.07–1.54)	2.25 (0.89–5.67)	0.28 (0.06–1.42)	1.95 (0.74–5.14)
Cultural identity						
Turkish			referent	referent	referent	referent
German, German-Turk^a^			0.73 (0.21–2.51)	2.24* (1.15–4.38)	0.94 (0.26–3.41)	2.02* (1.01–4.07)
Other identity			1.26 (0.40–3.98)	1.16 (0.48–2.84)	1.65 (0.49–5.55)	1.12 (0.45–2.79)
Mother tongue						
Turkish			referent	referent	referent	referent
German, both			0.22 (0.03–1.80)	0.87 (0.30–2.55)	0.21 (0.02–1.81)	0.86 (0.29–2.55)
Other language			0.69 (0.08–6.00)	0.81 (0.21–3.20)	0.71 (0.08–6.65)	.87 (0.21–3.59)
Language proficency						
Turkish			referent	referent	referent	referent
German, both			1.59 (0.18–14.22)	1.25 (0.45–3.53)	1.79 (0.18–18.28)	1.12 (0.39–3.28)
Length of residency, y						
German-born					referent	referent
**≤** 10					2.52 (0.41–15.56)	1.03 (0.18–5.78)
10–20					0.61 (0.09–4.12)	1.85 (0.60–5.75)
21–30					1.54 (0.25–9.48)	1.65 (0.54–5.06)
> 30					1.42 (0.27–7-51)	1.41 (0.70–2.83)
Citizenship						
Turkish					referent	referent
German, both					0.53 (0.16–1.70)	1.32 (0.66–2.67)

^a^or person with Turkish migration background. *p < 0.05. Model fit:

Model 1: LR _male_ = 5.19(6), *p* = .520; *R* ^*2*^_male_^=^ .05 LR _female_ = 7.84(6), *p* = .291; *R* ^*2*^_*female*_^=^ .04,

Model 2: LR _male_ = 9.07(11), *p* = .615; *R* ^*2*^_male_^=^ .09, LR _female_ = 13.88(11), *p* = .240; *R* ^*2*^_*female*_^=^ .07,

Model 3: LR _male_ = 12.40(16), *p* = .716; *R* ^*2*^_male_ = .12, LR _female_ = 17.26(16), *p* = .369; *R* ^*2*^_*female*_ = .09

**Table 4:** Risk factors of 12-month prevalence rates of dysthymia – OR and 95% CI

	Dysthymia					
	Model 1		Model 2		Model 3	
	Men	Women	Men	Women	Men	Women
Age, y						
18–29	referent	referent	referent	referent	referent	referent
30–49	1.82 (0.49–6.75)	1.15 (0.48–2.75)	3.40 (0.79–14.56)	1.08 (0.45–2.59)	6.60* (1.30–33.49)	1.46 (0.56–3.83)
50–65	3.50 (0.90–13.63)	1.75 (0.65–4.70)	6.48* (1.39–30.23)	1.66 (0.61–4.49)	29.42**(3.74–231.17)	2.41 (0.71–8.26)
Education						
High	referent	referent	referent	referent	referent	referent
Moderate	0.91 (0.30–2.74)	1.62 (0.64–4.07)	0.64 (0.20–2.06)	1.59 (0.62–4.04)	0.88 (0.25–3.14)	1.71 (0.65–4.53)
Low	1.54 (0.61–3.88)	2.23 (0.99–4.99)	1.59 (0.60–4.25)	2.12 (0.93–4.83)	2.94 (0.96–8.95)	2.56* (1.05–6.20)
Income, €						
> 1418	referent	referent	referent	referent	referent	referent
922–1417	1.39 (0.31–6.26)	2.47 (0.49–12.41)	1.73 (0.34–8.18)	2.59 (0.51–13.27)	1.55 (0.28–8.61)	2.55 (0.49–13.23)
**≤** 921	2.01 (0.53–7.55)	2.67 (0.60–11.98)	2.74 (0.65–11.58)	02.59 (0.57–11.87)	3.25 (0.71–14.86)	2.71 (0.58–12.76)
Cultural identity						
Turkish			referent	referent	referent	referent
German, German-Turk^a^			2.06 (0.75–5.66)	0.56 (0.27–1.17)	2.18 (0.72–6.58)	0.50 (0.23–1.09)
Other identity			1.72 (0.57–5.19)	0.91 (0.40–2.11)	1.75 (0.54–5.72)	0.90 (0.38–2.14)
Mother tongue						
Turkish			referent	referent	referent	referent
German, both			3.68* (1.24–10.29)	0.83 (0.23–3.01)	3.26 (0.97–11.00)	0.76 (0.20–2.83)
Other language			dropped	0.94 (0.24–3.69)	dropped	0.88 (0.22–3.61)
Language proficency						
Turkish			referent	referent	referent	referent
German, both			0.41 (0.09–1.91)	0.69 (0.30–1.59)	0.69 (0.13–3.67)	0.69 (0.28–1.67)
Length of residency, y						
German-born					referent	referent
**≤** 10					1.92 (0.36–10.32)	1.08 (0.29–4.05)
10–20					0.63 (0.12–3.18)	0.77(0.27–2.20)
21–30					0.61 (0.13–2.87)	0.43 (0.14–1.35)
> 30					0.18 (0.04–0.81)	0.66 (0.23–1.86)
Citizenship						
Turkish					referent	referent
German, both					2.69 (0.91–7.99)	1.60 (0.75–3.40)

^a^or person with Turkish migration background. ‘dropped’ refers to no cases found. *p < 0.05, **p < 0.01. Model fit:

Model 1: LR _male =_ 10.12(6), *p* = .120; *R* ^*2*^_male_ = .08, LR _female_ = 13.094(6), *p* = .057; *R* ^*2*^_*female*_ = .06,

Model2: LR _male =_ 23.72(11), *p* = .014; *R* ^*2*^_male_^=^ .19, LR _female_ = 16.26(11), *p* = .132; *R* ^*2*^_*female =*_ .08,

Model 3: LR _male =_ 33.32(16), *p* < 0.05; *R* ^*2*^_male_ = .26, LR _female_ = 19.13(16), *p* = .262; *R* ^*2*^_*female*_ = .10

According to Table 3, only cultural identity was a significant predictor of the prevalence of MDD among female participants. In Model 3, when adjusted for the confounding factor of migration status, female individuals who described their cultural identity as German, German-Turk, person of Turkish migration background or other cultural identity showed a risk about double as high for MDD relative to individuals with a Turkish cultural identity (*p* < 0.05).

In Table 4, it can be observed that higher rates of dysthymia were related to older age among male participants, in particular (*p* < .05) when controlled for acculturation status. Additionally, when adjusted for migration status, older male subjects were almost 30 times more likely to suffer from dysthymia than their counterparts in the young age group (*p* < .001). Compared to male participants who stated Turkish as their single mother tongue male participants claiming German and Turkish as their mother tongue showed a higher risk of dysthymia (*p* < .05). Significance was lost however when controlled for migration status. When controlled for the confounding variables of acculturation and migration status, female participants with a low education level were more likely to suffer from dysthymia (*p* < .05).

Symptom severity (mild, moderate or severe) of MDD was significantly associated with gender, χ^2^ (*N* = 662) =8.53, *p* < .05. Moderate symptom severity was observed in 70.3% of the women suffering from MDD compared to 29.7% among men. Similarly, 74.5% of women with MDD showed severe symptoms in comparison to 25.5% among men. Other risk factors did not show any significant association with the symptom severity of MDD.

## Discussion

### Key findings

The most important finding of this paper is that 12-month prevalence rates of depressive disorders (including MDD and dysthymia) among the study participants are very high with a minimum of 14.4% for MDD and 29.0% for any depressive disorder. By comparison with a recent study of the German native population and a meta-analysis of European studies, 12-month prevalence rates of diagnosed depression were at 6% for the German population and 6.9% for the population of the European Union [52, 53]. Higher prevalence of depression among Turkish migrants of both migration generations compared to the host population and other migrant groups has been found in previous population-based studies [26–31]. This paper demonstrates that individuals of older age and individuals with low socioeconomic status in particular display high prevalence rates of depressive disorders. Associations found between sociodemographic, acculturation-related and migration-related risk factors and depressive disorders are complex and partially different for male and female participants, as earlier European and American studies suggested [23, 28]. Results concerning symptom severity of MDD also show a relationship with gender. Moderate to severe symptoms of MDD are most prevalent among women.

Consistent with previous research studies of Turkish migrants, other migrant populations and the German native population, the strongest and most consistent independent predictor of increased risk of depressive disorders is older age among both genders [12, 29, 52]. The experience of migration and psychosocial factors were found to be related to higher depression rates in elderly migrants [12, 29]. This association is most present for dysthymia alone, which might be explained by a higher probability of diagnosing a persistent depressive disorder in older persons. A higher level of income is connected to lower prevalence rates of any depressive disorder among male participants, whereas among female participants, reduced risk of dysthymia is associated with a high educational level. This might derive from diverging role allocations in Turkish culture, since men may possess a stronger responsibility to provide for the family income. Lower socioeconomic status only partly explains higher prevalence rates of depressive disorders, which was also concluded in preceding studies of depression among Turkish and other migrants in Europe [10, 26, 27, 29].

Results concerning cultural identity and mother tongue are not consistent and have to be interpreted with caution. A trend can be observed that individuals who orient themselves towards the Turkish culture of origin and the German host culture simultaneously as indicated by their proficiency of both languages are at higher risk of depressive disorders. Previous studies reported contradictory results concerning individuals with Turkish migration backgrounds. Integration, which implies an orientation towards both cultures, correlated with lower prevalence rates of depression [38, 39]. Values and cultural-associated behaviours and practices labelled as typically Turkish or German might collide to an extent and, rather than fostering any benefits, may cause individuals more psychological distress.

Migration status shows minor confounding effects for sociodemographic variables but does not demonstrate any independent association with prevalence rates of depressive disorders. It can be argued that measures of migration status have been studied more widely among American and Canadian studies, which found strong associations between migration status and depression [6–8]. Surprisingly, risk factors of depressive disorder did not differ between migration generations in this sample as preceding studies of individuals with Turkish migration backgrounds have demonstrated [27, 38].

Results of the applied proxy measures of predicted variance limited the explanatory power of the models, which indicates that the assessed risk factors do not predict the prevalence of depressive disorders sufficiently [51] and should be interpreted with caution. One might be tempted to assume that Turkish individuals are generally more affected by higher prevalence rates of depression compared to Germans, but an international epidemiological study could not report significantly higher prevalence rates of depressive symptoms among Turkish compared to people of other nationalities, including Germany [54]. A possible explanation might be the selectivity of the Turkish migrant population, which initially immigrated as labour migrants to Germany. Further research studies should try to identify risk factors which apply more accurately to individuals with Turkish migration backgrounds in Germany in order to explain the high rates of depressive disorders. An interesting aspect would be to differentiate between different motives of migration within and between migrant groups, which might be related to the risk of developing depressive symptoms. Additionally, it might be useful to consider discrimination as a risk factor because evidence was found that it was related to poor adaptation among young Turkish migrants and higher depression rates among different migrant groups in Europe [10, 55]. Furthermore, the distinction between sexes when it comes to subtypes of depressive disorders is emphasised as this paper observed differences in their relationship with different risk factors.

### Strengths and Limitations

This paper provides important epidemiological data on Germany’s largest migrant group in a non-clinical setting. The exploration of the influence of migration and acculturation status is an important contribution towards a better understanding of what causes higher prevalence rates among individuals with Turkish migration backgrounds. Notably, differentiating between males and females in the analysis allowed for an in-depth examination of associations with various risk factors in both groups as gender has been identified as an important variable related to depression in different migrant groups as well as in the native German population [23, 28, 29, 38, 52].

Another strength of this paper lies in the strong effort that was made to reach individuals with Turkish migration backgrounds by carrying out a pilot study to analyse potential barriers of participation for individuals with Turkish migration backgrounds [43]. Extensive recruitment of participants was conducted by identifying individuals of the first and second generation via the onomastic procedure, snow ball sampling and on-site recruitment accompanied by a public media campaign. The relatively low response rate of individuals with Turkish migration backgrounds despite all the aforementioned efforts is an indicator of the constricted reachability of the target group and is in line with previous findings [29].

A further advantage of this study is that all survey material was available in Turkish and German and internationally standardised interviews were conducted in the language of choice by bilingual trained interviewers. The use of standardised diagnostic instruments to assess depression among non-western migrant groups was supported by a study, which demonstrated that depressive symptoms profiles were equally associated with functional impairment across different migrant groups, including Turkish migrants [56].

However, it has to be mentioned that only a limited number of risk factors have been measured and other factors have to be identified to predict prevalence rates of depression more precisely. The indicators used for acculturation do not cover all aspects of the complex multi-dimensional acculturation process such as cultural values or behaviour in culturally relevant situations. It should be known that indicators such as language proficiency are used as a proxy measure of acculturation which could capture different relations than intended. Nevertheless, meta-analyses found that most acculturation instruments were primarily based on the measurement of linguistic elements [19, 21]. Furthermore, the self-report of language proficiency by study participants is likely to limit the accuracy of the measure compared to standardised measures of language proficiency. Given that about 70% of study participants chose to be interviewed in Turkish we can assume that Turkish language proficiency is most likely represented more accurately than the self-reported ability to speak German.

An additional limitation of the paper lies in the representativeness of the sample, which is restricted by the differing recruitment procedures of the two recruitment centres and the location of the recruitment in two of Germany’s biggest cities in specific areas with a high density of individuals with Turkish migration backgrounds. Moreover, the possibility of differing prevalence rates between the study samples in Berlin and Hamburg should be considered in future studies. A further critique relates to the measures, which had to be taken such as the application of a quota scheme to enhance the representativeness of the study and the use of a monetary incentive to increase participation in the study. An additional selection effect may have occurred because participants who had already been affected by psychiatric symptoms or disorders may have been more likely to participate in this study than healthy individuals.

Another limitation of this paper relates to the differing group sizes and the lack of data variability, which constrained the data analysis and reduced the power of the models. The informative value of the conclusions was limited by merging categories together in order to perform statistical analysis. Furthermore, the first generation is overrepresented compared to the second generation which might have led to a bias towards higher prevalence rates of depression, as recent studies reported that the first generation of Turkish migrants showed higher rates of depression [27, 38].

## Conclusion

Individuals of Turkish migration backgrounds show high prevalence rates of depressive disorders, which are associated with older age and partly with low socioeconomic status. Symptom severity of MDD correlates with gender, with severe symptoms of this appearing mainly in female participants. Acculturation in the sense of an orientation towards culture of origin and host culture is related to higher prevalence of subtypes of depression. The need for more representative studies of individuals with Turkish migration backgrounds is emphasised along with further exploration of risk factors. Despite the findings that individuals with Turkish migration backgrounds in Germany are at a high risk of suffering from depressive disorders, migrants are underrepresented in the German outpatient mental health care system [57]. It exists a need for an extended focus on protective factors and barriers within the mental health care system in order to develop policies for prevention and intervention programs for individuals with Turkish migration backgrounds in order to facilitate equal access to health information and mental health service.

## Abbreviations

CI: Confidence Intervals
CIDI DIA-X: Composite International Diagnostic Interview Diagnostic Expert system for disorders
DSM-IV-TR: Diagnostic and Statistical Manual of Mental Disorders-IV-Text Revision
ICD-10: International Classification of Diseases-10
LR: Likelihood Ratio
M-CIDI: Munich Composite International Diagnostic Interview
OECD: Organisation for Economic Cooperation and Development
OR: Odds Ratio
SPSS: Statistic and Analysis Software
